# The economic burden of coronavirus disease 2019 (COVID-19): evidence from Iran

**DOI:** 10.1186/s12913-021-06126-8

**Published:** 2021-02-11

**Authors:** Mohsen Ghaffari Darab, Khosro Keshavarz, Elnaz Sadeghi, Javad Shahmohamadi, Zahra Kavosi

**Affiliations:** 1 School of Management and Medical Informatics, Shiraz University of Medical Sciences, Shiraz, Iran; 2 Health Human Resources Research Centre, School of Management and Medical Informatics, Shiraz University of Medical Sciences, Shiraz, Iran; 3 Student Research Committee, School of Management and Medical Informatics, Shiraz University of Medical Sciences, Shiraz, Iran; 4 Student Research Committee, Shiraz University of Medical Sciences, Shiraz, Iran

**Keywords:** COVID-19, Coronavirus, Economic burden, Health policy, Hospital costs, Intensive care units, Cost-of-illness, Iran

## Abstract

**Abstract:**

This study aimed to estimate both direct medical and indirect costs of treating the Coronavirus disease 2019 (COVID-19) from a societal perspective in the patients at a referral hospital in Fars province as well as the economic burden of COVID-19 in Iran in 2020.

**Methods:**

This study is a partial economic evaluation and a cross-sectional cost-description study conducted based on the data of the COVID-19 patients referred to a referral university hospital in Fars province between March and July 2020. The data were collected by examining the patients’ records and accounting information systems. The subjects included all the inpatients with COVID-19 (477 individuals) who admitted to the medical centre during the 4 months. Bottom-up costing (also called micro-costing approach), incidence-based and income-based human capital approaches were used as the main methodological features of this study.

**Results:**

The direct medical costs were estimated to be 28,240,025,968 Rials ($ 1,791,172) in total with mean cost of 59,203,409 Rials ($ 3755) per person (SD = 4684 $/ 73,855,161 Rials) in which significant part (41%) was that of intensive and general care beds (11,596,217,487 Rials equal to $ 735,510 (M = 24,310,728 Rials or $ 1542, SD = 34,184,949 Rials or $ 2168(. The second to which were the costs of medicines and medical consumables (28%). The mean indirect costs, including income loss due to premature death, economic production loss due to hospitalization and job absenteeism during recovery course were estimated to be 129,870,974 Rials ($ 11,634) per person. Furthermore, the economic burden of the disease in the country for inpatient cases with the definitive diagnosis was 22,688,925,933,095 Rials equal to $ 1,439,083,784.

**Conclusion:**

The results of this study showed that the severe status of the disease would bring about the extremely high cost of illness in this case. It is estimated that the high prevalence rate of COVID-19 has been imposing a heavy economic burden on the country and health system directly that may result in rationing or painful cost-control approaches.

## Background

The novel coronavirus disease, currently known as Coronavirus disease 2019 or simply COVID-19 (caused by the SARS-CoV-2 pathogen), was first introduced as an epidemic in December 2019 in Wuhan, Hubei, China [1, 2]. It was increasingly spread in many countries on all five continents of the world, with the World Health Organization (WHO) announcing in March 2020 that the COVID-19 pandemic was one of the health issues raising international concern [3]. The WHO also called for early diagnosis, isolation and treatment of the patients, contact tracing, and social distancing in order to break the chain of virus transmission [4].

According to the statistics published on the worldometers database, more than 16,433,715 million people in the world had been infected with COVID-19 by July 2020, of whom over 652,454 individuals have lost their lives. The information of this database also showed 291,172 and 15,700 for these two figures about Iran, respectively [5]. Transmission of the virus through respiratory droplets has caused the disease to spread rapidly and infect many people [6]. Individuals with a mean age of 55.5 years [7] and those with underlying conditions, such as hypertension, diabetes, and heart disease, are at a higher risk [8, 9]. Thus, the health status of the elderly and those with underlying illnesses are twice as much important because demand aggregation followed by the collapse of the health system may prevent the provision of routine medical services to the people in need. For example, modelling studies by the World Health Organization showed that stopping the provision of antiretroviral therapy services in the Sub-Saharan could lead to an additional 500,000 AIDS-related deaths [10, 11].

From an economic perspective, the spread of COVID-19, the ever-increasing number of patients, and the complications of the disease have imposed high direct medical and indirect costs on patients, the health system and the government [12]. As far as the economic burden of direct medical costs is concerned, although the costs vary with the number of the infected people, the severity of the disease, mean length of stay in the hospital, mean length of stay in the ICU, and other factors [13, 14], international studies showed that medical costs of the patients with COVID-19 were significantly higher than those of other infectious diseases due to the higher probability of hospitalization and mortality [15]. These circumstances are also right about the need for COVID-19 patients for special care services and the related costs [16–18]. Statistics have shown that 3% of the people with COVID-19 in the world are in critical condition and need special care services [5]. The results of a study on 138 individuals with COVID-19 in Wuhan, China, indicated that 26.1% of the patients used special care services, of whom 41.6 and 47.2% received non-invasive and invasive ventilation, respectively [19]. In Lombardy, Italy, 88% of the patients admitted to the ICU received invasive ventilation, and 11% received non-invasive one [20]. The daily cost of hospitalization in the ICU is generally 3–4 times higher than in general wards [21].

Moreover, the lost income due to the disease or mandatory stay at home or the death of the workforce due to the disease could be considered as indirect costs [22–24]. The prevalence of COVID-19 and closure of many industries would reduce Gross Domestic Product (GDP) [25, 26]. According to studies, the global spread of the disease will lead to a decrease in global GDP by 2.5–3% per month [27]. Furthermore, the outbreak of COVID-19 severely damaged the world’s top fifteen economies [28]. Comparing the economic effects of this disease with those of past epidemics shows that the economic crisis facing the world is much more severe than what was experienced in the past.

Therefore, regarding the high costs of the treatment of the patients with COVID-19 and its resulting economic burden on the health system and the country generally, and due to the impending economic crisis, it seems crucial to calculate direct medical and indirect costs of treatment of the disease.

## Methods

This applied research is a partial economic evaluation and a cross-sectional cost-description study. The research population consisted of all the patients with COVID-19 who had referred to a primary referral medical centre for COVID-19 patients in Fars province, Iran, by July 2020. As the research sample was equal to the research population, no sampling was done, and all the patients were examined through a census.

This included cost and demographic information of all confirmed cases undergoing the treatment processes from diagnosis to discharge at *the Hospital*. The patients whose part of the treatment process had been performed outside *the hospital* and were referred to other hospitals or a rehabilitation centre were excluded from the study. The required data were collected through the forms designed in two sections: the patient’s demographic information and characteristics, and cost information. Given that the present study was conducted from the society perspective, the costs included direct medical (DC) and indirect costs (IC). Based on a set of measures, public policies and health protocols related to unprecedented circumstances of disease control and prevention in the time of COVID-19, people have been encouraged to keep physical and social distance and avoided having unnecessary social contacts, we restricted the direct cost estimation to medical cost only. In other words, there were little possibilities for interviewing patients in order to gather relevant information. It was going to put people at a lower risk of illness. Further, there was no access to an alternative source of information to collect such data.

The bottom-up costing method was used to calculate direct medical costs due to the lack of a proper and accurate database. The bottom-up methodology uses comprehensive information on procedure circumstances and input utilization data from reports (or observed usage) related to each patient at the service provider level to calculate cost per unit. Indeed, it is based on collecting all individual data of consumption of resources and then aggregating all individual costs, summing them to achieve the total costs. Although bottom-up approach might be more time-consuming, it is comparatively more accurate as well [29].

The incidence approach was also used to provide cost information [30]. There may be little difference between the prevalence and incidence approach in this study, significantly, given that COVID-19 is an emergent disease which there have not been long-term sequelae associated with it yet. However, since this approach is more practical in the case where it will help policy-makers to develop preventive measures and while we included patients from the onset of this epidemy in Iran, it seems that this term properly matches to the methods here [31]. The solution to incidence is based on the idea that a stream of health-related expenses should be allocated to the year in which the stream begins. Thus the approach to incidence allocates all direct, morbidity and mortality costs based on their present-value and to the year in which the disease first appears [32, 33].

### Calculation of direct medical costs

In this study, the calculation of direct medical costs conducted based on electronic medical data through the hospital information system (HIS). The data, subsequently, were classified into nine categories as follows: the costs of intensive and ordinary bed services, physician visit cost_ in Iran’s health system cost of the visit includes the fee that is paid for each time a physician a patient at their bedside to examine or prescribe them_, counselling, nursing services, drugs and medical consumables, rehabilitation, dialysis, imaging, electrography, laboratory, and other services (including monitoring in non-intensive care units, compilation and prescription of therapeutic diets, cannula implantation for venous-venous-hemodialysis, blood and blood products transfusion, tracheal intubation, level-one critical care or triage, blood oximetry or continuous pulse oximetry, temporary catheter insertion into the bladder, and cold room). The average total direct medical costs of each patient with COVID-19 were calculated as the sum of the average inpatient cost in each category:

**Table:** 

**Total direct medical cost per patient =** (the mean cost of counselling) + (the mean cost of nursing services) + (the mean cost of medicine and medical consumables) + (the mean cost of rehabilitation) + (the mean cost of dialysis) + (the mean cost of imaging) + (the mean cost of electrography) + (the mean cost of laboratory) + (the mean cost of other items)	

Regarding the significance of investigating the cost difference between the inpatient care for severe cases needing care in ICU or otherwise, direct medical cost for those patients was calculated and reported separately. This also is able to demonstrate the variability among the quantities. Service Charges used in calculation and estimations of this study all are based on official prices which had been approved by health authorities at the national level.

### Calculation of indirect costs

In this study, the indirect costs were also investigated based on the income-based human capital approach and measured in two parts, including the wages that a patient loses due to absenteeism (hospitalization or home rehabilitation) and the productivity loss as a result of a patient’s premature death at the ages of 15 to 65. The lost productivity was considered zero for the individuals under the age of 15 and over 65 who were not economically productive.

The Forgone Labor Output (FLO) equation was used to calculate the potential economic production loss due to the premature deaths:

P_l_ = $$ {\sum}_{i=1}^N\frac{W\ast {\left(1+g\right)}^i}{{\left(1+r\right)}^{i.}} $$

In this equation, P_l_ is the current value of the predicted future income per workforce, N is the mean working years per person at the age of i, W shows the mean current income per person, r stands for the social discount rate, and g is the annual real income growth rate per person [34].

Economic Burden:

$$ Total\;\cos t\;\left( direct\kern0.17em medical\;\cos t+ indirect\;\cos t\right)\times estimated\kern0.17em number\kern0.17em of \operatorname {inf} ected\kern0.17em inpatient\kern0.17em cases\kern0.17em in $$

In order to estimate the total economic burden on the country, the total cost (total direct medical and indirect costs) for each patient was first calculated and then multiplied by the total number of cases with COVID-19 throughout the country [35].

Since the available data suggested that the disease lasted for an acute episode and less than 30 days, the discount rate was not applied; in addition, all the costs were calculated based on purchasing power parity (PPP) 2020, adjusted by, equivalent to 15,766 Rials per 1 dollar [36].

## Results

### Epidemiological finding

Of the 477 patients studied, none of the patients was under 15 years old, 41% were 15–45 years old, 38% were 45–65, and 21% were 65 years or more; Among them, 56% were male, and 44% were female.

(Table 1: Table 1: Demographic and Disease Severity Information of the Patients). Fever, muscle pain, cough and respiratory distress were the main signs and symptoms among the patients. Nearly half of the patients suffered from at least one of the underlying diseases diabetes, heart disease or hypertension. Oxygen saturation levels were below 93% in 41% of the patients. 4% received tracheal intubation. Moreover, 7% of the patients received ICU care due to a severe form of the disease.

**Table 1:** Demographic and Disease Severity Information of the Patients

Gender	Variable	Total number	%
	Male	266	56
	Female	211	44
**Age Groups**	0–15	0	0
	15–45	197	41
	45–65	180	38
	+ 65	100	21
**Severe Cases** **(ICU patients)**	**(ICU patients)**	36	7

### Direct medical costs

According to the results, the total direct medical costs were estimated to be 28,240,025,968 Rials ($ 1,791,172), in which the intensive and general care beds expenses were the largest share of costs (41%) with 11,596,217,487 Rials equals to $ 735,510. Following that, the main costs were those of medicines and medical consumables (28%) with 8,044,070,257 Rials equals to $ 510,209 physician visits costs (12%) 3,422,848,975 Rials equal to $ 217,100, and electrography and laboratory (9%) 2,645,752,049 Rials equal to $ 167,811. Nursing and consulting services each one accounted for merely 2% of total costs (Table 2: Medical Direct and Indirect Costs of studied patients).

**Table 2:** Indirect and Direct Medical Costs of studied patients

Direct Medical Cost	%	Total cost all patients	Mean cost per patient	SD	Mean Cost Per non-sever patient	SD	Mean Cost Per ICU patient	SD
General and Intensive Care Beds	41	11,596,217,487 (IR) 735,510 ($)	24,310,728 (IR) 1542 ($)	34,184,949 (IR) 2168 ($)	18,466,239 (IR) 1171 ($)	18,490,652 (IR) 1173 ($)	95,905,725 (IR) 6083 ($)	69,111,570 (IR) 4384 **($)**
Physician Visit Costs	12	3,422,848,975 (IR) 217,100 ($)	7,175,784 (IR) 455 ($)	3,810,035 (IR) 242 ($)	7,037,094(IR) 446 ($)	3,465,158 (IR) 220 ($)	8,874,734 (IR) 563 ($)	6,422,523 (IR) 407 **($)**
Consultant and surgeon	2	544,642,687 (IR) 34,545 ($)	1,141,809 (IR) 72 ($)	1,469,135 (IR) 93 ($)	940,031 (IR) 60 ($)	1,138,595 (IR) 72 ($)	3,613,588 (IR) 229 ($)	2,218,364 (IR) 141 **($)**
Nursing services	2	597,734,159 (IR) 37,912 ($)	1,253,111(IR) 79 ($)	1,449,037 (IR) 92 ($)	1,014,794 (IR) 64 ($)	828,864 (IR) 53 ($)	4,172,504 (IR) 265 ($)	2,926,280 (IR) 186 **($)**
Drugs and supplies	28	8,044,070,257 (IR) 510,209 ($)	16,863,879 (IR) 1070 ($)	30,468,242 (IR) 1933 ($)	12,396,651 (IR) 786 ($)	17,803,503 (IR) 1129 ($)	71,587,424 (IR) 4541 ($)	68,004,175 (IR) 4313 **($)**
Rehabilitation and Dialysis	1	358,674,154 (IR) 22,750 ($)	751,937 (IR) 48 ($)	2,897,736 (IR) 184 ($)	373,737(IR) 23 ($)	1,587,375 (IR) 101 ($)	5,384,891 (IR) 342 ($)	7,226,606 (IR) 458 **($)**
Imaging	2	704,609,641 (IR) 44,691 ($)	1,477,169 (IR) 94 ($)	1,191,832 (IR) 76 ($)	1,368,356(IR) 87 ($)	1,045,763 (IR) 66 ($)	2,810,123 (IR) 178 ($)	1,786,642 (IR) 113 **($)**
Electrography and Laboratory	9	2,645,752,049 (IR) 167,811 ($)	5,546,650 (IR) 352 ($)	5,263,980 (IR) 334 ($)	4,729,388 (IR) 300 ($)	3,672,308 (IR) 233 ($)	15,558,103 (IR) 987 ($)	9,015,815 (IR) 572 **($)**
Other services	1	325,476,558 (IR) 20,644 ($)	682,341 (IR) 43 ($)	459,965 (IR) 29 ($)	635,339 (IR) 40 ($)	383,438 (IR) 24 ($)	1,258,107 (IR) 80 ($)	781,671 (IR) 50
**Total**	**100**	**28,240,025,968 (IR) 1,791,172 ($)**	**59,203,409 (IR) 3755 ($)**	**73,855,161 (IR) 4684 ($)**	**46,961,630 (IR) 2979 ($)**	**41,459,732 (IR) 2630 ($)**	**209,165,200 (IR) 13,267 ($)**	**151,535,892 (IR) 9611 ($)**
**Indirect Cost**			**Mean cost per patient**					
Lost income (hospitalization)			5,950,524 (IR) 378 ($)					
Lost income (recovery at home)			16,800,000 (IR) 1065 ($)					
Potential productivity Loss (premature death)			107,107,949 (IR) 10,190 ($)					
**Total**			**129,870,974 (IR) 11,634 ($)**					

### Indirect costs

#### Productivity loss due to job absenteeism

Of the patients treated, 6.5% died; of these, 4% were in productivity age, who died an average of 11 years before age 65. The results showed that the mean length of hospital stay for each COVID-19 patient was 7 days on average (max = 38, min = 2) (this figure for sever cases was 11 days on average with max and min of 38 and 2, respectively); concerning this, the total lost income of a COVID-19 patient at the age of 15–65 due to hospitalization was estimated to be 5,950,524 Rials (equal to $ 378) (According to the data of the Statistical Centre of Iran, the minimum monthly wage in 2019 was 24,000,000 Rials, equal to the daily income of 800,000 Rials per individual).

Also, based on the epidemiological data, a patient’s lost income due to absenteeism during the recovery period of 21 days was estimated to be 16,800,000 Rials (equal to $ 1065) (Table 2: Medical Direct and Indirect Costs of studied patients).

### Production loss due to premature death

Estimation of Potential Production Loss due to premature death followed a methodology previously described based on the Forgone Labor Output equation (section 2: Method). Regarding this, average lost years was calculated 11 years based on deducing the age at death from expected working age (65 years) in patients between 15 and 65 years of age. Due to the lack of detailed wage information, the minimum annual wage was considered as the average income per capita in 2020 equal to 288,000,000 Rials (based on information from the ministry of cooperative labour and social welfare), and the annual growth rate for real income per capita (g) was considered 3%. The discount rate also was set at 6%. Finally, the economic loss caused by every premature death from COVID-19 estimated to be 2,677,698,726 Rials (equal to $ 169,838).

P_l_ = $$ {\sum}_{i=1}^{11}\frac{\mathrm{288,000,000}\ast {\left(1+0.03\right)}^i}{{\left(1+0.06\right)}^i} $$ = 2,677,698,726 Rials

According to the mortality rate among hospitalized cases derived from the study, only 4% of this amount (129,870,974 Rials equal to $ 11,634) was considered in the calculation of the average indirect costs per inpatient.

Economic burden imposed on all inpatients with COVID-19 in Iran estimated to be 22,688,925,933,095 Rials equal to $ 1,439,083,784 in the country based on the number of all inpatient cases equal to 120,000 individuals up until the end of July and the average cost of disease;

## Discussion

This study was the first study on the cost of illness among COVID-19 patients in Iran. The outbreak of the COVID-19 and the increasing number of patients in Iran imposed high costs on the infected people and the health system. The economic burden of the disease in addition to the economic crisis facing the country certainly due to economic sanctions imposed on Iran by the United States have been causing concerns among the managers and policy-makers in the health sector. Hence, identifying the economic consequences of COVID-19 can be valuable evidence for policy-making.

The average direct medical costs for these patients were estimated to be $ 3755 per person. Based on a recent study in the United States conducted by Bartsch et al., the average direct medical costs per person were estimated to be $3045 throughout the infection [15]. In this regard, the results of these two studies are comparable. The results of this study also showed that this record of information varied with the severity of the disease. Critically-ill patients who received ICU services accounted for the largest share of direct medical costs. Although nearly 7% of the patients with COVID-19 had received ICU services, they accounted for almost 26% (7,529,947,215 rials/$ 477,600) of the total direct medical costs. Indeed, on average, patient care in intensive care units was four times more expensive than other patients.

The spread of the disease caused not only direct costs but also many indirect ones, including the potential production loss due to job absenteeism during hospitalization and recovery course at home, as well as production loss due to premature death. The productivity loss of the patients with COVID-19 was about two times more than the direct medical costs per patient on average.

According to the findings of this study, the mean direct and indirect costs in total certainly for inpatient cases was 43,173,908,604,828 Rials so far, which was accounting for 1.5% of the average annual health expenditures in 2018. Public hospitals in Iran’s health system are funded through two primary sources including the Social Security Organisation (IHIO) reimbursement based on fee-for-service (FFS) and per-diem payments or the government budget by the Ministry of Health and Medical Education (MoHME) [37]. Furthermore, patients need to pay 10% of expenses for hospital services [38]. This also is applying to care and services in the case of COVID-19. Nonetheless, in order to determine whether this condition may impose catastrophic costs on patients and their families, more studies are needed to examine the issue through considering household incomes and expenses and patients’ capacity to pay for treatment.

Recent experiences have shown that in the absence of an effective treatment or vaccine, limiting the working hours and activities of small businesses or promoting telecommuting in the context of social distancing have been used as the main measures related to the control and prevention of COVID-19.

Although the resulting recession could pose additional economic challenges to government funding for these jobs, given the contagious nature of the disease, the epidemic, and the high prevalence rates _that can lead to disruption in routine health care as well as specific care provision for COVID-19_ and also with respect to the results of this study based on the high costs of this disease for various sectors including the health system, investing in creating distance working possibilities and considering livelihood support packages as incentives that people are determined to follow the protocols, can be offset by reduction in the financial burden of the disease, including the financial burden of premature death and reducing treatment costs.

### Research limitations and suggestions for future studies

Due to the limited access to socioeconomic information of the participants in this study, complementary investigation of the work productivity loss by the individuals’ income deciles was not carried out. Another research limitation was the unprecedented circumstances during the COVID-19, which avoid researchers conducting an in-depth investigation of indirect cost through an interview with patients and non-medical direct cost estimation such as travel expenses save for productivity loss. Therefore, considering the mentioned issues suggested in future studies.

## Conclusion

Examining the economic dimensions of the disease through this research provided significant evidence for making socioeconomic policies related to COVID-19 and similar epidemics. According to the results of this study, the high rate of pathogenicity, a considerable percentage of patients in need of intensive care and subsequently the enormous costs of the disease (direct and indirect) can face the country’s health system with unprecedented and significant economic stress in financing government and university medical centres. Thus, these centres might be forced to apply painful cost-control and rationing policies in providing the necessary care to this group of patients and others.

## Data Availability

The datasets used and analyzed during the current study are available from the corresponding author on reasonable request.

## Abbreviations

COVID-19: Corona Virus Disease 2019
DC: Direct Cost
FLO: The Forgone Labor Output
GDP: Gross Domestic Product
IC: Indirect Cost
ICU: Intensive Care Unit
PPP: Purchasing Power Parity
WHO: World Health Organization
